# Statistics and sovereignty: the workings of biopower in epidemiology

**DOI:** 10.3402/gha.v8.28262

**Published:** 2015-06-22

**Authors:** Amand Führer, Friederike Eichner

**Affiliations:** Independent Scholar, Halle/Saale, Germany

Sifting through recent public health literature, an urgent need and an intense enthusiasm for the opportunities of civil and vital registration become apparent (1–3). Without intending to question the validity of the arguments calling for the improvement of civil registration in low-income countries, we want to take the opportunity to elucidate a critical perspective on registration-related issues, which draws on long-established debates in the social sciences.

The national census performed in Uganda in August 2014 illustrates our first criticism. In line with international scientific and political arguments in favor of civil registration, the Ugandan government emphasized the great importance of robust data for budget allocation and (among other issues) health planning (4). The census was presented as a chance to update statistics and thus improve Uganda's position in discussions with international donors (5). However, while the enumerators were roaming the country, almost every day, national newspapers reported that people were trying to evade the census, with some even resorting to violent means to disturb enumeration (6, 7). A number of religious leaders who rejected the census for spiritual reasons were arrested and charged for sabotage, while the enumeration of their followers was enforced by armed police (8, 9). Clearly, not everybody was happy about being granted the right to be counted.

From the perspective of a social scientist, this is by no means surprising. After all, considering the cultural appropriateness of surveys as well as taking into account the possible misuses of registration data has been a topic of discussion in the biomedical literature (10). Meanwhile, our argument aims to make a more general point: we want to consider some of the implications which epidemiologic work carries with regard to the sociological notion of biopower.

First made popular by Michel Foucault, the term biopower defines those forms of power that aim to control, regulate, and monitor a population's biologic life and subject it to governmental interventions (11). The health of individuals and populations becomes a concern of politics. Taking a historical perspective as the starting point, Foucault contrasts power in the modern state to power in the premodern state, which considered the sporadic enforcement of unrestricted and arbitrary power as its right. The premodern sovereign's prerogative was death due to his right to take life (in the form of punishment, torture, or execution), but he did not have power over everyday life (12). In contrast, power in modern states has great bearing on everyday life and is centered around questions concerning life, not death. Life as biological fact thus became an object of power. This transformation was facilitated by the emergence of the notion of population, which created the foundation to regulate and control life. Through surveillance of ‘[p]ropagation, births and mortality, the level of health, life expectancy and longevity, with all the conditions that can cause these to vary’, the optimization of life was made possible (13).

While Foucault conceptualizes the liberal state as the principal agent of biopower, the situation in many African countries is constituted in curiously different ways, with non-governmental organizations being the most important governing bodies in many areas (14). NGO staff and medical personnel, in particular, therefore assume the position of the sovereign with all its problems, and public health can be read as means to discipline society.

Here we want to further exemplify this point by considering the particular intersections of biopower and epidemiology: As Nguyen illustrates in his book, the epidemiologist's job can be described as ‘naming, mapping, grouping’ (15). Apparently an innocent activity, the identification of risk groups and target groups for interventions becomes problematic once one realizes that epidemiology is by no means purely descriptive: in contrast, often the groups identified in statistical analyses are not ‘naturally’ existing groups, but are created by the very process of their epidemiological identification. Thus, epidemiologic research is a productive process that has the potential to create social identities and realities. In their studies in the Ukraine after the disaster in Chernobyl and the growth of the AIDS epidemic in West Africa in the 1990s, Petryna (16) and Nguyen (15) coined the term biologic and therapeutic citizenship. They observed how membership in an epidemiologically defined group allowed people to access crucial infrastructure. Even though access to this infrastructure (such as social security funds and health care) is *de jure* open to every citizen, in fact only those affected by an epidemiologically noted biologic defect qualify for access. Excluding everybody not fitting into relevant categories, the paradox arises that ‘sometimes the only way to survive is by having a fatal illness’ (15).

Thus, a census as mentioned at the beginning does not only have the potential to provide a basis for the improvement of healthcare services, human rights, etc. but also bears the danger of excluding those who are not willing to participate or those whose needs are not covered adequately in the survey instrument from access to these very services. Failing to qualify for biologic/therapeutic citizenship might, therefore, lead to the *de facto* loss of *any meaningful* citizenship.

The discussion of biopower should by no means be misinterpreted as an argument against epidemiological research. But, in the same way as the physicians are aware of the potential side effects of drugs they prescribe, the epidemiologists and the public health community at large should be aware of the potential side effects of their work. The awareness that statistical analyses might create new social identities should inform the design of epidemiological studies and censuses while from a Foucauldian perspective, public health activities need to reflect on their inherent power over life.

*Amand Führer*
Independent Scholar
Halle/Saale
Germany
E-mail: amand.x@gmx.net *Friederike Eichner*
Independent Scholar
Halle/Saale
Germany
